# Do the parents of the youth abusing substances need to be supported? A literature review study

**DOI:** 10.4102/curationis.v46i1.2401

**Published:** 2023-02-20

**Authors:** Lina S. Hlahla, Charity Ngoatle, Tebogo M. Mothiba

**Affiliations:** 1 Department of Nursing, Faculty of Health Sciences, University of Limpopo, Polokwane, South Africa

**Keywords:** Parents, youth, support, substance, abuse

## Abstract

**Background:**

Substance abuse negatively affects the youth who use substances, their families and especially their parents. The use of substances impairs the health of the youth and is linked to an increase in noncommunicable diseases. Parents become stressed and they need help. Parents fail to carry out daily plans and routines because they are not sure what the substance abuser can do or what can happen to the substance abuser. When the parents’ well-being is taken care of, they will be able to take care of their youth when they need help. Unfortunately, little is known about the psychosocial needs of the parents, especially when their child abuses substances.

**Objectives:**

This article aims to review the literature to explore the need for support for parents of youth abusing substances.

**Method:**

The study adopted the narrative literature review (NLR) methodology. Literature was retrieved from the following databases and search engines: electronic databases, search engines and hand searches.

**Results:**

Substance abuse has been found to affect the youth abusing substances and their families negatively. The parents, being the most affected, need support. The involvement of health professionals can assist the parents in feeling supported.

**Conclusion:**

Parents need support programmes that will give support and strength to their existing abilities.

**Contribution:**

Focusing on the support needs of the parents of youth abusing substances will help to ensure parents are supported and mentally healthy.

## Introduction

The reality is that substance abuse affects millions of parents across the world (Meadows 2016). When one child abuses substances, the whole family becomes affected. Parents feel overwhelmed by their child’s diagnosis and find it difficult to process. Siblings may experience sleep disturbances, and their attitudes may change, especially if the parents channel all their attention to the child who abuses substances (Jones et al. 2019).

Substance abuse negatively affects the youth who use substances, their families and their communities. The use of substances impairs young people’s health and is linked to increased physical and psychological disorders. These effects are also visible in their family members. The abuse of substances by adolescents exposes them to violent crimes as victims or perpetrators. They will be in conflict with the law and may suffer loss of employment (Moore 2017). The use of substances by the youth leaves an emotional wound on the families and the parents. Parents find it difficult to watch their children being in danger of long-term unemployment because they drop out of school, are ostracised from the community and are dangerous to themselves and others because of substances (DSD 2013; Meadows 2016).

Dealing with a youth who drinks or uses drugs can limit the time and attention parents give to their other children at home. Feeling ignored, the siblings may act out as a means of being noticed. An older sibling may also be a negative role model for the younger youth. The younger child may engage in alcohol or drug use to be like the older sibling (Moore 2017).

The impact of youth substance abuse causes a significant change in the family cycle. In addition, there is a severe conflict between the restructuring and readjustments to the new realities (Breiner et al. 2016). A family is a social group that determines the individual’s formation and development in the affective, cognitive and psychological domains (Clark, Donnellan & Robins 2016). However, the whole family changes when a child is using substances and is addicted because they must make new arrangements, add and create new expectations and have new realities (Martinsa et al. 2015).

Hospitalisation because of substance abuse can be demanding for the substance abuser and their families. The family will need to frequently visit the hospital to check on their beloved family member, as they want to know how they are recovering (Root et al. 2016). The parents must understand what is expected of them during their child’s hospitalisation, as this will help in lowering distress and helping the parents to be there for their youth and maintain a key role in their duties (Fixter et al. 2017).

## Methodology

The literature review methodology adopted for this study is the narrative literature review (NLR). Narrative literature review is a method of review that assists the reviewer to identify, assess, analyse and interpret the body of knowledge on the support needed by the parents of youth abusing substances. It places and rationalises the choice of the topic within the framework of existing literature and finds gaps in existing knowledge (Coughlan & Cronin 2016). Narrative literature review allowed the reviewers to find literature from diverse sources. The reviewers control the literature available to determine what they need for the study. The reviewers can easily control what they want by choosing the databases, key search terms and combining terms to suit what they want, time limits and limitations of language. The reviewers outlined the findings regarding the themes developed from the literature (Coughlan & Cronin 2016). Going through the literature assisted the reviewers in acquiring knowledge on the studied topic.

### Database searches

Literature was retrieved from the following databases and search engines:

- Electronic databases: Ethno Med, Public Library of Science (PLOS ONE), British Medical Journal (BMJ) Open, BioMed Central (BMC), Sabinet and ScienceDirect.
- Search engines: Google Scholar, University of Limpopo (UL) E-Libraries, Chrome and Google Books.
- Hand searches: Reference lists from retrieved literature.

### Key search terms

The keywords used in the literature search were multiple combinations of ‘substance abuse’, ‘youth and substance abuse in youth’, ‘substance abuse in adolescents’, ‘experiences of parents in substance abuse in youth’, ‘substance abuse effects in the family’, ‘parenting a substance abuser’, ‘parents needs about substance abuse’, ‘support needs of parents’, ‘effects of substance abuse on the parents’, ‘contributory factors to substance abuse’.

### Parameters

Only publications meeting the following criteria were included in the literature review:

- full articles related to support of parents of youth abusing substances
- English publications available by the time of the literature review
- articles published from 2012 to July 2022.

The literature review excluded articles that were not written in English.

### Review process

Two review mechanisms were considered in this analysis. The first review entailed locating the relevant studies and screening. The screened publications were re-evaluated following the predetermined eligibility criteria in the second review. The publications were found using a variety of electronic databases.

Table 1 gives a brief summary of the literature as it was searched. The literature was searched from the search engines, databases and hand searches, producing 1050 articles. Moreover, a total of 750 publications remained after the duplications were removed. Furthermore, 554 publications remained after the researchers screened the abstracts. The second review was performed on the remaining publications. The researchers rescreened the publications. The rescreening of publications produced 330 publications. The reassessment for the second review produced 198 publications; as others did, they addressed other substance abuse issues not related to support for parents of youth abusing substances. A further 155 publications were excluded or eliminated because they did not meet the eligibility criteria. After the review process, 51 articles were included in this review. Table 1 illustrates the outcome of the search.

**TABLE 1:** Review process.

Databases searched	Total publications
**First review**	
Identification of publications	1 050
Publications remaining after duplications were removed	750
Excluded publications after screening abstracts	554
**Second review**	
Publications remaining after rescreening	330
Publications assessed for the second review	198
Publications remaining after 151 articles excluded	51
Overall publications used in the study	47
Included publications in this review	20

The findings of the literature search are summarised as follows: 51 sources were reviewed for the study literature, including websites, journals and books.

## Findings

The review of the literature was performed and yielded the following themes:

- the effects of substance abuse on the youth
- the effects of substance abuse on the siblings
- the effects of substance abuse on the family
- the effects of substance abuse on parents
- the need to support the parents
- the healthcare professionals’ involvement
- the need for a support programme.

Table 2 outlines the themes and references used during literature review.

**TABLE 2:** Themes and references.

Theme	References
The effects of substance abuse on the youth	Hartney 2018 Eds. Kaye, Vadivelu & Urman 2014 Ewing et al. 2015 Moore 2017
The effects of substance abuse on the siblings	Morrill et al. 2018 Moore 2017 Ye et al. 2017
The effects of substance abuse on the family	Morrill et al. 2018 Moore 2017 Dykes & Casker 2021
The effects of substance abuse on parents	Tollefson et al. 2017 Gadsden, Ford & Breiner 2016 Purcell et al. 2018 Cousino & Hazen 2013 Conn et al. 2018
The need to support the parents	Curtis et al. 2016 Gates et al. 2019 Wiseman et al. 2018 Both et al. 2018
The healthcare professionals’ involvement	Golsäter et al. 2016 Nightingale et al. 2017 Dunst & Trivette 2014
The need for a support programme	Dunst & Trivette 2014 Bray et al. 2017 Smith 2018

### The effects of substance abuse on the youth

The literature review has indicated that substance abuse affects the youth. The indicators of substance abuse include taking more substances for a more extended period than the person wants; using substances continuously even when one knows it puts their life in danger and affects their physical and mental health; having a need to stop using the substance but failing; using more of the time in trying to acquire, use or recover from substance abuse; having solid cravings for substances; failing to do what one is supposed to do at home, work, or school because of use of substances; ongoing use, even though it creates social problems; not enjoying what one used to do before starting to use substances; requiring more substances to feel good and developing withdrawal symptoms, which need one to take more of the substance (Hartney 2018; eds. Kaye et al. 2014). The use of substances by the youth is associated with adverse effects such as not progressing well at school, risky sexual behaviours, delinquency and continued use, which can lead to future alcohol and drug use and concurrent mental and physical problems (Ewing et al. 2015). Some behaviours that can be noticed when a person abuses substances can be mood swings, increased argumentativeness and secretiveness or withdrawal from the family. These factors can disrupt normal functioning between the parent and the youth at home, leading to increased disagreements or fights. The side effects of the abuse of substances can be conflicts with the parent over money, school and friends (Moore 2017).

### The effects of substance abuse on siblings

The literature review has indicated that siblings can also be affected when their fellow sibling abuses substances. The use of substances by one sibling may lead to other children or siblings missing out on the attention and time of their parents. If the parents are not careful, the other children will start to build resentment towards them and their substance-abusing siblings. Commonly, most of the attention will be given to one child without considering the effects the status quo has on the health of the other family members (Morrill et al. 2018).

Dealing with a teen who drinks or uses drugs can limit parents’ time and attention to their other children at home. Feeling ignored, those children may act out as a means of being noticed. An older sibling may also be a negative role model for the younger youth. The younger child may engage in alcohol or drug use to be like the older sibling (Moore 2017). The whole family becomes both physically and psychologically affected when one member abuses substances. When a child abuses substances, the parents carry the burden of caring for that child. In addition, the parents fear losing their child; they experience negative emotions. The siblings are not given the attention their substance-abusing siblings receive from their parents. The whole family may become depressed and in need of help. They need interventions to improve their well-being, psychologically and physically (Ye et al. 2017).

### The effects of substance abuse on the family

The use of substances by a family member has an impact on the whole family; however, the impact varies according to family members. Some effects include unmet developmental requirements, poor attachment, financial difficulties, legal issues, emotional pain and even violence (Dykes & Casker 2021). Some of the parents feel like the condition of their youth at home keeps their family hostage. One child’s condition can affect the whole family, including the siblings. There is a need for the family to cancel some family vacations to attend to the substance abuser. Parents fail to carry out daily plans and routines because they are not sure what the substance abuser can do or what can happen to the substance abuser (Morrill et al. 2018). Substance abuse by the youth has financial implications for the family (Moore 2017).

### The effects of substance abuse on parents

Many parents feel depressed and anxious because of their child’s substance abuse. They compromise their activities, including their families, to accommodate the affected child. They may also put their careers on hold. This leads to poverty, as poverty constitutes a risk for parenting. The lack of finances in the family will affect the ability of parents to give food, keep their youth healthy and take them to good schools. Parenting at this stage becomes more complex; the life of compromising even their happiness becomes a regular part of their lives (Gadsden et al. 2016; Tollefson et al. 2017).

According to Purcell et al. (2018), when parents care for their youth, they may experience challenges because of their medical encounters. Parents find themselves going through both emotional and physical stressors when providing care for their youth. For example, mothers reported feeling unsure about their capability to manage symptoms. They said they do not know how to balance action and safety, and they do not know which signs and symptoms to report to members of the healthcare team or the police following their youth’s hospitalisation. Parents also reported increased guilt, worry, anxiety and fear levels. Parents also acknowledged that they do not have the psychosocial strategies to deal with these negative emotions. According to Cousino and Hazen (2013), the use of substances by youth hurts the parents’ life. The parents suffer the impact of their child abusing substances as much as their child suffers the effects of substance abuse. Parents have a role in assisting their substance-abusing children even though they are not skilled. These demands lead to parents’ poor psychological and physical health. They end up not being able to manage their own lives and their own families. Conn et al. (2018) state that parenting a child who abuses substances increases parental stress and the risk of poor parenting and future behavioural problems by other children. Parents need emotional support to deal with the adverse effects of parenting stress.

### The need to support the parents

Parents are essential in the recovery of their youth. The parent’s ability to cope with the stress associated with substance abuse in their youth can affect the family’s quality of life (Curtis et al. 2016). When admitted, parents want to know what is happening with their child but often feel neglected by the healthcare workers. They feel that healthcare providers do not provide timely information and support. They experience fears when they feel uninformed about what is happening to their admitted child. Thus, the professionals need to know the timing, the source and the type of information they should give the parents (Gates et al. 2019). When the parents’ well-being is taken care of, they will be able to take care of their youth when they need help. Unfortunately, little is known about the psychosocial needs of the parents, especially when their child abusing substances is admitted to the hospital (Wiseman et al. 2018). When parents do not receive enough support from healthcare providers, they lose confidence in the healthcare system. This leads to the underutilisation of available healthcare services. Therefore, parents must be attended to in the healthcare facilities. This will help relieve parental stress and assist in family adaptation (Both et al. 2018).

### The healthcare professionals’ involvement

There is a need for healthcare providers to be involved and assist the parents with coping strategies (Dunst & Trivette 2014). As healthcare providers, nurses have a role in supporting the whole family of the admitted patients. The nurse can hold meetings with the family, which could be for a short time, like 15 min. That time can be enough for nurses to empower the relatives of the patients with knowledge and understanding based on the family situation to provide the needed support for family members (Golsäter et al. 2016). Parents may often feel overwhelmed and isolated. They look up to the healthcare professional to assist with care. This means that the healthcare professionals (nurses, social workers, psychologists, psychiatrists) must meet the admitted youth’s clinical needs and the parents’ educative role. The healthcare professional can support the parents by asking them what they need to learn and their preferences regarding the kind of support they need. However, at times there are no existing validated interventions for health professionals to utilise when making assessments for the learning needs and preferences of the parents (Nightingale et al. 2017).

### The need for a support programme

Parents need support programmes that will give support and strength to their existing abilities. The support programmes can encourage the development of new capabilities so that parents will know and have the skills needed to perform child-rearing responsibilities efficiently and give their youth opportunities and experiences that will endorse learning and development (Dunst & Trivette 2014). Support programmes give the parents the ability to give an account of their worries and anxieties and with other parents who had the same experiences and have been through a similar situation. What other parents have been through is the most important distinguishing factor of support the parents may need. Parents need to be in contact with the parents who managed to escape the stress of managing a child who abuses substances, which will help them to grow and have meaningful relationships with their youth (Bray et al. 2017). The support programmes can be also planned to accommodate the whole family (Smith 2018).

## Discussion

Substance abuse is a disease that kills individuals, harms families and cripples society. When youth engage in substance abuse, the parents as primary caregivers will be affected by the situation. This is because parents expect only the best out of their youth from birth. One thing that does not cross the parents’ minds is that their youth can abuse substances at some point in their lives (Meadows 2016). While parents are expecting their youth to have a bright future, there are possibilities that they will grow up and become addicted to substances or alcohol. Substance abuse may seem like a distant problem that is far off, a problem that may never affect the parents or the family, but in real life, it devastatingly affects family life. The reality is that substance abuse affects millions of parents across the world (Meadows 2016).

The use of substances by the youth is a challenge; this is because, according to Dykes and Casker (2021), youth take drugs more than any other age group. This typically occurs as a part of a young person’s growth stage when they pass from adolescence to young adulthood. This is because, as people mature to become independent and autonomous, the brain develops in the early twenties. Some cultural and societal conventions that favour substantial alcohol use are part of adolescent life. For whatever reasons, the parents of youth who abuse substances end up at the receiving end when their children become addicted or are hospitalised because of substances. Parents are under pressure to display competent parenting (Li & Song 2022).

The family and the siblings are affected when one family member abuses substances. The use of substances by an adolescent member of the family can leave unaddressed bitterness and anger from other siblings. This is because the parents may shift their attention from their other children to focus on the child who now uses substances. This may cause the parents to feel inadequate and can lead to parent dissatisfaction (Dykes & Casker 2021). Social support is essential for the well-being of the parents, as it gives them satisfaction and boosts their confidence and morale. Parents rely on their families for help when their youth abuse substances. Poor family cohesion may lead to increased parental stress and dissatisfaction. Successful parenting comprises cooperation and support from the immediate family network. Family as an organisation is characterised by mutual support, stability and communication among members (Li & Song 2022).

The parents must make sacrifices for their youth. Some of the sacrifices the parents go through include financial sacrifices. They take the little money they have to finance the well-being of their child by paying hospital bills or rehabilitation centres. They even go to the extent of battling with medical aid companies to ensure that their youth’s healthcare needs are met. This leads to incredible frustration as the parents have to assume new roles such as nursing their youth by making sure that they take the medications as they are supposed to and also assuming the role of being a psychologist to the child (Meyers 2015).

Parents are essential to family life. Decent parenting can promote well-being, health and physical and emotional development of the family, in addition to prohibiting ill health in succeeding generations. Parents’ confidence in managing their children, especially youth, determines a healthier quality of family life (Moen, Opheim & Trollvik 2019). Parents of youth abusing substances know what it is like to be strong, weak, brave and terrified. They experience a spectrum of emotions that most parents will never need to know. Their capacity to love and care is tested to the limit (Brisson 2016).

Most parents who come to the hospital bringing their youths who abuse substances have low health literacy, making it difficult for them to make health decisions for their admitted child. Health literacy occurs when the individual can obtain, process and comprehend the simple health information and services needed to make suitable health decisions. It is difficult for adults with low health literacy to understand what is required to obtain maximum health for their children; they sometimes make wrong treatment decisions. Low health literacy leads to parents bringing their youth to the hospital when the situation is worse (May et al. 2018).

In the study carried out by Sim et al. (2018), parents expressed anger for not being found in need of help when they bring their youth to the hospital. They felt humiliated by the hospitals because no communication was made to them on how the treatment was being given to their youth. The parents felt distressed because they felt powerless and had no hope for the future of their youth. Their stress was caused by the fact that they were overwhelmed by their youth’s ill health and mental instability, and sometimes they failed to cope. Some parents said that many things have changed in their lives as a result of their youth’s sickness because of substance abuse. They suffered symptoms such as forgetfulness and confusion. They did not know how to deal with their youth’s ill health; they felt like they lost their child daily (Sim et al. 2018).

Mothers, as the parents of youth abusing substances, are most affected by substance abuse of their youth. According to Xu et al. (2018), mothers can go through what is called maternal parenting stress. This maternal parenting stress comprises the experiences and perceptions of the mothers when they have faced difficulties meeting their expectations and requirements because of insufficient social and personal resources to deal with household demands. Maternal parenting stress negatively affects mothers and youth regarding behavioural, social and psychological outcomes. Maternal parenting stress is related to mothers’ lowered life satisfaction and mental health problems, family fights, poor attachment with the youth and sometimes even the maltreatment of the children. Different child factors are associated with maternal parenting stress. These factors include health, temperament, gender and age. Mothers become more stressed when a girl child abuses substances than a boy child. This could be because it is socially expected that a boy child is often problematic (Xu et al. 2018).

Parents reported that they have high stress levels and receive little help regarding this from healthcare providers. The stress levels of the parents increase as their children grow into adulthood. The first manifestation of abuse of substances in the family leads to the process of family adaptation. Whether the youth can adapt adequately to their recovery process from substance abuse also depends on the ability of their family to cope with the stress caused by substance abuse (Both et al. 2018). Parenting stress can increase because the parent has a child with social-emotional problems. Problems such as poor parenting and child behavioural problems are common when the parent is distressed. The parents need to be equipped with interpersonal skills so that they are to manage the challenges that come with parenting. The parents may benefit from learning more about substance abuse and how it impacts their youth’s development, including socio-emotional and neurobiological impacts (Conn et al. 2018).

Parenting stress is common and challenging to parents of all types and structures of families. The stress can even be high for the caregivers of substance abusers. At the same time, there may be other children in the house who are younger and need the intervention and attention of the parent. The parents feel a loss of a child who abuses substances because they were born ordinary but may have mental health problems because of substance abuse. There may be experiences of family conflicts, and financial strains may result from the youth abusing substances (Richardson et al. 2018). These challenges give rise to parenting stress. They lead to problems with parenting and other relationships in the home, such as the coparenting relationship. It was found that the mothers who reported high levels of parenting stress also suffered poorer coparenting relationships with their partners (Richardson et al. 2018).

Some of the challenges faced by the parents include having to deal with the diagnosis of their youth, being able to manage youth abusing substances from day-to-day or additional need, being able to maintain family life and commitments at work and having to deal with unforeseen changes in their youths’ condition and family circumstances (Bray et al. 2017). When a child is hospitalised because of substance abuse, the parents of such a child constantly interact with the hospital staff. They have more questions than answers about the prognosis of their child. They find themselves forced to decide carefully on their child’s treatment. They see the hospital as a stressful environment, influencing the parents’ reaction towards hospitalising their admitted child. They can even be scared if their child’s condition worsens. At this point, they have to deal with their mental health and work on improving their coping strategies. They must ensure that their family is functioning well even when the other member demands attention in the hospital (Foster et al. 2017).

When parents lack knowledge about their child’s condition, they do not become very involved in caring for their youth. They fear complications for their child, which limits the involvement of the parents in caring for the child. There is a need for healthcare providers to be involved and assist the parents with coping strategies. When the nurses do not interact with the parents, the latter are discouraged and afraid to ask questions. Nurses and other health professionals appear to allocate care duties to them without adequate explanation. When a professional fails to give the information to the parent, it is the same as denying the parents an opportunity to care for their youth (Valizadeh, Ahmad & Zarea 2016).

The parents have indicated that they need to be informed about health matters relating to their admitted youth. They need this information to understand what is going on with their youth and what needs to be performed as a way forward. In the process of being informed, they also want their families to be involved collectively to assist each other in helping the admitted family member. They need family intervention therapies instead of individual ones to deal with the problem of their youth being admitted for substance abuse (Gates et al. 2019).

Parents need to know about parental monitoring, that is, knowing where their children are at a particular time and what they are doing. That way, the parents will have an idea of the whereabouts of their youth, the activities they are engaging in and how they are coping. This means that the parents will be intentional in the care of their children, and they will be able to seek information about any change in their youth. When the parents are aware of their youth’s movements, it will be easy for them to notice any difference in behaviour, and they will be able to immediately seek help (Hernandez, Rodriguez & Spirito 2015).

Botzet et al. (2019), in their study, have shown that if parents can have solid parent–adolescent bonds, there will be a reduction in the likelihood of adolescent drug use. Similarly, authoritative parenting and the topics parents can raise, such as monitoring, the rule set, communication and guided experience, influence how youth internalise parents’ attitudes, values, beliefs and health behaviours, including substance use. Most importantly, it is more effective for parents to communicate with their children and tell them what is expected of them while they are still young so they may grow up knowing what the parents want.

The parents want to know about their youth when their children abuse substances and are admitted to the hospital. They want to know if they could one day quit substances. Furthermore, they want to know if there is anything they could do to help their young ones abstain from substances, and they also want to know how they will live when they are at home after being admitted. They must be equipped with skills to manage their youth, especially at home after discharge (Nightingale, Friedl & Swallow 2015).

Parents suffer anxiety and fear about the illness of their admitted children. They may feel emotionally exhausted to have a child in the hospital. Some feel guilty, particularly when their adolescent is admitted for substance abuse (Golsäter et al. 2016). The parents’ priority is to learn about their youth’s condition; they want to know about treatment and daily management. This information gives them confidence and helps them feel in control and adjust to the future. Parents seek information so that they are able to answer people who may have questions. However, the parents do not always receive the information they want from healthcare providers. Sometimes the healthcare providers do not cover all the aspects of the information needed by the parents (Nightingale et al. 2015).

Healthcare professionals are experts, from the parents’ view. They are the experts in the hospital system and medical care of their youth. Parents appreciate the expertise of healthcare professionals; hence, they try to build the parent–healthcare professional relationships around the medical care of the admitted youth. The relationship between parents and healthcare professionals involves two key behaviours, namely learning how things work and ensuring survival (Butler, Copnell & Helen 2019). Parents therefore look to healthcare professionals for support. However, the nurses feel the parents need to be involved in their children’s well-being when in the hospital. The nurses believe that it is necessary for the parents to know about their children’s condition and to keep on being updated on their developments so that they can be involved (Golsäter et al. 2016).

The healthcare provider has to support the parents, but they sometimes fail to provide needed support to the parents because of the high workload. Participation of the parents in the delivery of care is a well-recognised way of engaging the parents. However, there is a need to understand the family’s needs during the youth’s admission. Nurses find it challenging to deal with both the patient and the parent. Parents, on the other hand, find themselves lacking confidence in communicating with healthcare professionals (Curtis et al. 2016). Nurses should therefore collaborate with other healthcare professionals in meeting the needs of the parents. Furthermore, Behrndt (2015) states that it is hard to support people through difficult times. It may feel confusing for the healthcare professional, especially because they must show empathy to the parents of youth abusing substances. The healthcare professional may experience much confusion when placed in a situation of supporting a parent of a substance abuser and also having to manage the substance abuser on the other hand.

There are other behaviours that parents need to learn to cooperate with healthcare professionals. They need to learn how to step back and allow healthcare professionals to do their work. They must learn to accept the restrictions and defer to medical advice. They also need to acknowledge that their skills are limited. Otherwise, most parents know that they have limited medical knowledge to make informed decisions regarding the care of their admitted youth. They know that they have no idea of examinations that need to be performed and the risks involved. As parents do not know much about medical procedures, they often submit to the advice given by medical practitioners to make sure their youth receive the relevant prescription (Butler et al. 2019).

Support programmes can be necessary for the parents as they provide the parents with the ability to give an account of their worries and anxieties and engage with other parents who had the same experiences and have been through a similar situation. What other parents have been through is the most important distinguishing factor of support the parents may need. Parents need to be in contact with the parents who have the same problem of children abusing substances to learn the ways of managing a child who abuses substances, which will help them to grow and have meaningful relationships with their youth (Bray et al. 2017).

Support programmes for the parents may include topics such as overall access for families, support for families from an early stage and the family’s involvement at every level of operation of the programme. Parenting programmes sometimes include different parenting activities such as parent information classes and parent–child sessions and support sessions, which include parenting materials. Individualised supports for parents are provided in response to specific child-rearing problems or particular questions that the parents can raise. This will provide, or help parents to access, different kinds of resources and supports, such as childcare resources and medical resources (Dunst & Trivette 2014).

Smith (2018) states that there is a need to implement family-centred care. Executing family-centred care will benefit nurses, families and the youth. For the youth’s outcomes to improve and for the family stress to be reduced, there is a need for open communication, mutual respect and working together to develop, deliver and evaluate patient care. Family-centred care can assist nurses in putting together family-centred care values within the nursing practice.

Nightingale et al. (2015) support the conclusion that parents should be supported and taught about their youth’s needs. Parents have different ways of learning. They have different information and support and adapt differently to manage their youth’s condition. Various reasons explain why parents want information. The parent’s priority in most cases is to learn about the state of their youth, how to manage their youth daily and the treatment they have to give to this youth. That information reassures them and helps them plan for the future and be in control.

It is therefore essential for the nurse to acknowledge that the parents need to be informed, and they need to be reassured to have confidence in healthcare professionals providing care to their youth who is abusing substances. The nurses also need to be aware that parents have other needs that are related to their parenting roles that are not related to their admitted youth that are part of daily life, such as running a household, taking care of other family members and managing finances. Parents, on the other hand, need to know that the healthcare professionals are trustworthy and are concerned about their admitted youth (Feeg et al. 2018).

### Recommendations

For the parents of youth abusing substances to feel supported, the following steps are recommended.

For the parents:

- Parents must not hesitate to seek help for their youth abusing substances.
- They must also seek help for themselves so that they receive support while they are helping their youth who abuse substances.
- Parents need to be available when there are information sessions related to their youth abusing substances or any information related to substance abuse.

For the healthcare providers:

- As much attention is given to the youth abusing substances, further attention should be given to the parents so that they continue being healthy enough to take care of their child abusing substances.
- Healthcare providers should be available to share information with the parents; the information will help in reducing worries for the parents.
- Healthcare professionals can organise awareness programmes for the parents so that they know more about substance abuse and how to manage it.
- Workshops and support groups organised by the healthcare provider can assist the parents with relevant skills to manage their youth abusing substances.

## Conclusion

The findings of the review of literature have revealed that substance abuse has devastating effects on the youth abusing substances, their siblings, their families and the parents. Parents are found to be on the receiving end in all the effects, as they are supposed to show effective parenting to the whole family. Studies have shown that parents suffer emotional and physical health problems and they need support. One of the reasons they suffer is because they lack knowledge regarding substance abuse. It is essential that the health providers can come in to provide the needed support to the parents; parents can benefit from the support programmes that are tailor-made to enhance their knowledge of substance abuse. Further research is recommended on how to support the siblings and the whole family when one family member abuses substances.
